# The Role of Proline Rich Tyrosine Kinase 2 (Pyk2) on Cisplatin Resistance in Hepatocellular Carcinoma

**DOI:** 10.1371/journal.pone.0027362

**Published:** 2011-11-09

**Authors:** Wei Geng, Kevin T. P. Ng, Chris K. W. Sun, Wing Lung Yau, Xiao Bing Liu, Qiao Cheng, Ronnie T. P. Poon, Chung Mau Lo, Kwan Man, Sheung Tat Fan

**Affiliations:** Department of Surgery, The University of Hong Kong, Queen Mary Hospital, Hong Kong, China; Rush University Medical Center, United States of America

## Abstract

**Aims:**

*We previously demonstrated* Proline rich tyrosine kinase 2 (Pyk2) plays important roles in regulating tumor progression, migration and invasion in hepatocellular carcinoma (HCC). In this study, we aimed to examine the role of proline rich tyrosine kinase 2 (Pyk2) on cisplatin resistance in HCC and to explore its underlying molecular mechanism.

**Methodology/Principal Findings:**

Stable transfectants either overexpressing or suppressing Pyk2 were established in different HCC cell lines. MTT, colony formation and Annexin-V assays were employed to examine their *in vitro* responses to cisplatin. Xenograft ectopic and orthotopic nude mice models were generated to investigate the *in vivo* responses of them to cisplatin treatment. cDNA microarray was performed to identify Pyk2-induced genes which were further validated by quantitative real-time RT-PCR using clinical HCC samples. *In vitro* functional study demonstrated that Pyk2-overexpressing HCC transfectants exhibited relatively lower cytotoxicity, higher colony-forming ability and lower apoptosis to cisplatin compared with the control transfectants. Moreover, Pyk2 overexpressing HCC transfectants had a higher survival rate under cisplatin treatment by up-regulation of AKT phosphorylation. *In vivo* xenograft nude mice model demonstrated that Pyk2-overexpressing transfectants developed higher tolerance to cisplatin treatment together with less tumor necrosis and apoptosis. cDNA microarray analysis revealed that there were more than 4,000 genes differentially expressed upon overexpression of Pyk2. Several upregulated genes were found to be involved in drug resistance and invasion in cancers. Among them, the expression profiles of MDR1, GAGE1, STAT1 and MAP7 were significantly associated with the expression of Pyk2 in clinical HCC samples.

**Conclusions:**

Our results may suggest a new evidence of Pyk2 on promoting cisplatin resistance of HCC cells through preventing cell apoptosis, activation of AKT pathway and upregulation of drug resistant genes.

## Introduction

Hepatocellular carcinoma (HCC) is one of the most fatal diseases all over the world, particularly in developing countries[1]. Besides surgical treatments, systematic chemotherapy, play crucial roles in HCC treatment especially for patients with advanced HCC [2]. However, none of the single drug treatment strategies such as cisplatin, doxorubicin or 5-FU have shown significant survival benefit due to a high incidence rate of chemoresistance[2], [3]. Several potential molecular pathways in HCC are targeted for therapeutic interventions such as angiogenesis pathway, Raf/mitogen-activated protein kinase (MAPK)/extracellular signal-regulated kinase (ERK) pathways, epidermal growth factor receptor-1 (EGFR) and phospatidylinositol-3-kinase (PI3K)/AKT/mammalian target of rapamycin (mTOR) pathway and WNT/β-catanin pathway [4], [5], [6].Among those targeted agents, sorafenib, an oral multikinase inhibitor blocking tumor cell proliferation and angiogenesis, is the first agent to demonstrate a statistically significant improvement in the overall survival of HCC patients [7], [8]. However, most of the targeted agents demonstrated a very low response rate even for sorafenib [9]. So far, molecular mechanism of chemoresistance of HCC is not very clear, pushing an urgent need to seek for novel targets to understand and overcome this pressing issue.

Focal adhesion kinase (FAK) is a non-receptor tyrosine kinase key mediator of integrin-mediated signaling pathways that regulates cellular interactions with extracellular matrix [10], [11], [12]. Several lines of evidences suggested that FAK plays important roles in tumor initiation, progression and metastasis through manipulation of signaling pathways for survival, proliferation, migration, epithelial-mesenchymal transition, invasion, and angiogenesis [10], [11], [13]. Proline-rich tyrosine kinase (Pyk2) is a FAK related family member, sharing a 65% similarity in amino acid sequence and showing similar effect to FAK in regulation of cell motility and invasion [14], [15]. Pyk2 plays important parts in regulating the proliferation, differential and progression of human cancers including prostate cancer [16], [17], [18], lung cancer [19] and breast cancer [20], [21]. We previously found that there is significant correlation in the expression of FAK and Pyk2 in HCC patients [22]. FAK and Pyk2 are overexpressed in nearly 60% of tumor tissues of HCC patients. Moreover, overexpression of FAK in HCC patients is significantly correlated with larger tumor size, advanced new Edmonson's stage and shorter disease-free survival while positive overexpression of Pyk2 is significantly correlated with larger tumor size, advanced new Edmonson's stage, venous invasion, shorter overall survival and shorter disease-free survival, suggesting that Pyk2 is an important prognostic marker in addition to FAK [22]. Functional studies showed that Pyk2 promotes proliferation and invasiveness of HCCs through activation of c-Src and ERK/MAPK signaling pathways [23]. Furthermore, Pyk2 can promote motility of HCC cells through induction of epithelial to mesenchymal transition[24]. A recent study demonstrated that combination of sunitinib and a FAK/Pyk2 inhibitor (PF-562,271) effectively inhibits tumor angiogenesis and aggressiveness of human hepatoma in a rat xenograft model [25]. Based on the experimental and clinical evidence of Pyk2 on HCC progression and invasion, we hypothesize the possibility of Pyk2 on promoting drug resistance of HCC. In this study, we tried to investigate the role of Pyk2 in chemoresistance of HCC through a series of *in vitro* and *in vivo* functional studies.

## Materials and Methods

### Plasmids and antibodies

Plasmids pCDNA3-Pyk2 and pCDNA3-PRNK were gifts from Dr. Joseph Loftus, Mayo Clinic Scottsdale, USA. pCDNA3.1 (+) vector was purchased from Invitrogen (Carlsbad, CA). Monoclonal antibody against Pyk2 (clone 11) was purchased from BD Transduction Laboratories (San Jose, CA). Monoclonal antibody Phospho-Akt (Ser473) and Phospho-Akt (Thr308) were purchased from Cell Signaling (Danvers, MA). Rhodamine phalloidin, alexa fluor 488 goat anti-rabbit immunoglobulin G, alexa fluor 488 rabbit anti-mouse immunoglobulin G and rhodamine goat anti-rabbit immunoglobulin G antibodies were purchased from Molecular Probes (Carlsbad, CA).

### Chemotherapeutic drug (Cisplatin)

Cisplatin was manufactured in the Netherlands, Haarlem by Pharmachemie BV. The storage concentration is 1 mg/ml (50 ml = 50 mg) and kept at room temperature (15∼25°C).

### HCC patients, specimens and cell lines

Total RNAs were extracted from 43 pairs of HCC specimens (tumor and adjacent nontumorous liver tissues) collected at the Department of Surgery, Queen Mary Hospital, Hong Kong, China SAR. Clinical information was available from these 43 patients. All samples were anonymously coded according to the local ethical guidelines (as outlined by the Declaration of Helsinki), and informed written consents were obtained from all patients. The study was approved by the ethic committee of the University of Hong Kong.

Human HCC cell lines PLC and Hep3B were purchased from ATCC (Manassas, VA, USA) and were grown in Dulbecco's modified eagle medium containing 10% FBS, 2 mM L-glutamine, 100 units/ml of penicilium and streptomycin (Life Technologies, Carlsbad, CA). Human metastatic HCC cell line MHCC97L was a gift from Prof. Z.Y.Tang of Fudan University, Shanghai, China.

### Establishment of HCC stable transfectants

Cell transfection and isolation of stable transfectants were performed following the protocols reported previously[22]. Different protein expression patterns of Pyk2 in HCC cell lines were demonstrated in the previous study. Pyk2 expression level was significantly higher in Metastatic cell line MHCC97L by comparing with PLC and Hep3B. The full length of Pyk2 was transfected into PLC and Hep3B cell lines to increase the expression level of Pyk2. Meanwhile, the C-terminal of Pyk2, which was also called PRNK, was transfected into the MHCC97L cell line as a dominant negative to knock-down the Pyk2 expression level, whereas pCDNA3.1 (+) was transfected into these three HCC cell lines as a control.

### Western blot

Western blot assay was modified from a previous method [26]. Briefly, whole protein of stable transfectants was extracted using cell lysis buffer (Cell Signaling Technology, Beverly, MA). After protein concentration was determined by Bradford protein assay (BioRad, Hercules, CA), resolved proteins were transferred onto PVDF membranes (Amersham Biosciences, Piscataway, NJ) using mini Trans-blot cell (BioRad, Hercules, CA) with 1× transfer buffer at 4°C overnight. They were then incubated with corresponding primary antibodies at 4°C overnight. And then they were incubated with proper secondary antibodies. Finally, immune reactive signals were visualized by ECL Plus Western blotting Detection Reagents (GE Healthcare Bucks, UK). The molecular weights of the positive bands were determined by comparing their molecular weight with proper protein ladder (Fermentas, Gern Burnie, MD) on the membranes.

### Reverse Transcriptase-PCR

RT-PCR was performed to determine the mRNA level of the gene. Prior to RT-PCR analysis, contaminated genomic DNA in the RNA sample was removed by DNase I treatment (Invitrogen). Each of 1 µg RNA sample was treated with DNase I in a 10-µl reaction mixture containing 1 µl of DNase I (U/µl) and 1 µl of 10X DNase I buffer at room temperature for 10 minutes. One microliter of 25 mM EDTA was added followed by incubation at 65°C for 10 minutes. Each cDNA product was synthesized from 1 µg of RNA using the high capacity cDNA kit (Applied Biosystems, Foster City, CA, USA) under 25°C for 5 minutes and then 37°C for 2 hours. For cell line samples, PCR reaction for target genes including Pyk2, MDR1, GAGE, STAT1, Caspase9 and MAP7 was performed by using Taq PCR Kit (Promega, Madison, WI, USA) under the following PCR cycles: 95°C for 5 minutes, 35 cycles at 95°C for 1 minute, 57°C for 1 minute and 72°C for 1 minute. For internal control 18S, 30 PCR cycles were used. Primer sets used were described in Table 1.

**Table 1:** Sequence of primer pairs applied in this study.

Gene name	Sequences
Pyk2 (C-terminal)	Forward: 5′ CGGACTGATGACCTGGTGTA 3′
	Reverse: 5′ TTCTTCACCACCACCACGTA 3′
Pyk2 (N-terminal)	Forward: 5′ TGGGGAGGTCTATGAAGGTG 3′
	Reverse: 5′ ACTGCCTCGCTCATGAACTT 3′
MDR1	Forward: 5′ CAGAGGGGATGGCTAGTGTT 3′
	Reverse: 5′ CGTGGTGGCAAACAATACAG 3′
Casp9	Forward: 5′ CTAGTTTGCCCACACCCAGT 3′
	Reverse: 5′ ATGCGTCCAGGGTCTCAAC 3′
GAGE	Forward: 5′ CCGAAGCCTGAAGCTGATAG 3′
	Reverse: 5′ TTCACCTCCTCTGGATTTGG 3′
MAP7	Forward: 5′ CATGGCACAGCGAGCTATAA 3′
	Reverse: 5′ AGCTGGTTGTCTTGCTTTGG 3′
STAT1	Forward: 5′ TTCAGGAAGACCCAATCCAG 3′
	Reverse: 5′ TGAATATTCCCCGACTGAGC 3′

### MTT assay

MTT assay was performed to measure the number of viable cells under the cisplatin environment. Cells were trypsinized and counted by using a hemocytometer with 0.2% trypan blue (Life Technologies). Cells (5,000 cells) were seeded onto 96-well plates in normal culture condition for overnight. Cells were treated with different concentrations of cisplatin for 48 hours. MTT was added into each well and incubated at 37°C for 4 hours. Afterwards, 100 µl of MTT solvent was added into each well to dissolve the formed crystals. Signals were measured by a plate reader (BioRad). Each experiment consisted of four replications and at least three individual experiments were carried out.

### Colony formation assay

Colony formation assay was performed to investigate the effects of Pyk2 overexpression on cell proliferation under cisplatin environment. Cells (5×10^3^ cells/well) were seeded onto 6-well plates in normal condition for overnight. The cells were treated with different concentrations of cisplatin for 2 weeks. Culture medium was changed twice a week. After 2-week incubation, the colonies were washed with 1×PBS and fixed in 4% paraformaldehyde in 1×PBS. They were then stained with 0.5% crystal violet for 25 minutes at room temperature. The colonies were counted under a light microscope. A mean number of colonies were obtained from three independent experiments.

### Annexin-V-FLOUS assay

Annexin-V-FLOUS assay was applied to measure the percentage of apoptotic cells under the cisplatin treatment. Firstly, cells were seeded onto 6-well plates to 50% confluency. The cells were treated with different concentrations of cisplatin for 48 hours. Afterwards, thecells were trypsinized,collectedand washed with PBS. The cell pellet was suspended in 100 µl of Annexin-V-FLOUS and PI labeling solution (Annexin-V-FLOUS Staining Kit, Roche) and incubated for 10–15 minutes at 15–25°C. The stained cells were analyzed on the flow cytometer (FACSCalibur from BD, Franklin Lakes, NJ) by the CELLQuest software. Each experiment was analysed in triplicate and at least three independent experiments were performed.

### cDNA microarray analysis

cDNA microarray was performed following the protocols reported previously with some modifications[27]. Briefly, total RNAs of PLC-vector and PLC-Pyk2-8 transfectants were extracted by using the RNA spin mini kit (GE Healthcare, Bucks, UK). Genome-wide expression of human genes was examined with the GeneChip system Human Genome Microarray (Affymetrix, Santa Clara, CA). Microarray data were analyzed by GeneSpring version 7 (Silicon Genetics, Redwood City, CA). Genes with 2-fold changes in the expression level were selected and further analyzed with the software PathwayArchitect (Stratagene Corporation, La Jolla, CA). cDNA microarray data and the correlation with clinical samples were confirmed by SYBR Green real-time qRT-PCR. Primer designs are listed in Table 1.

### Orthotopic xenograft liver tumor model

The Orthotopic nude mice liver tumor model was established using MHCC97L-vector and MHCC97L-PRNK cells [28]. Briefly, approximately 1×10^7^ cells in 0.2 ml of a culture medium were injected subcutaneously into the right flank of Balb/c nude mice (male, 4 weeks old). The mice were observed daily for signs of tumor development. Once the subcutaneous tumor reached 1 cm in diameter, it was removed and cut into 1–2-mm cubes, which were then implanted into the left lobe of another group of nude mice (4 weeks old). Cisplatin treatment started 2 weeks after the implantation. Cisplatin (4 mg/kg) was injected intraperitoneally into the nude mice every 4 days and lasted for 4 weeks. The volume of liver tumors was determined according to the methods described by Janik *et al*.[29]. No less than six mice were performed for each group. The study had been licensed according to Animal (Control of Experiments) Ordinance Chapter 340 by the Department of Health, Hong Kong Special Administrative Region. (ref.: (08–490) in DH/HA&P/8/2/3 Pt. 7).

### Ectopic xenograft tumorigenesis model

Cells (1×10^6^/0.2 ml culture medium) were injected subcutaneously into the right flank of Balb/c nude mice (male, 4 weeks old). Once the diameter of the tumor reached 0.5 cm, the nude mice were treated by intraperitoneal injection of cisplatin (4 mg/kg) every 4 days for 4 weeks. The tumor size was recorded for 4-day intervals. After a 4-week treatment, the mice were sacrificed for tumor sample collection. No less than six mice were performed for each group. Animal studies had been licensed according to Animal (Control of Experiments) Ordinance Chapter 340 by the Department of Health, Hong Kong Special Administrative Region. (ref.: (08–490) in DH/HA&P/8/2/3 Pt. 7).

### Hematoxylin and eosin (H & E) and TUNEL staining

Tumor tissues were collected from both the ectopic xenograft tumorigenesis model and orthotopic xenograft liver tumor model. H & E staining was performed to display the nucleus and cytoplasm of the paraffin and frozen sections. TUNEL assay was performed to detect the apoptotic nuclei in paraffin sections by using *in situ* Cell Death Detection Kit(Roche Applied Science, Mannheim, Germany). These two assays were performed following the protocols reported previously [30].

### Statistical analyses

The 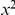 test was performed to compare discrete variables. Mann-Whitney *U*- test was used for statistical comparison of continuous variables. *P*<0.05 was considered statistically significant. Calculations were performed using the SPSS computer software version 15 (SPSS, Chicago, IL).

## Results

### Expression patterns of Pyk2 in HCC stable transfectants

**Figure 1:**
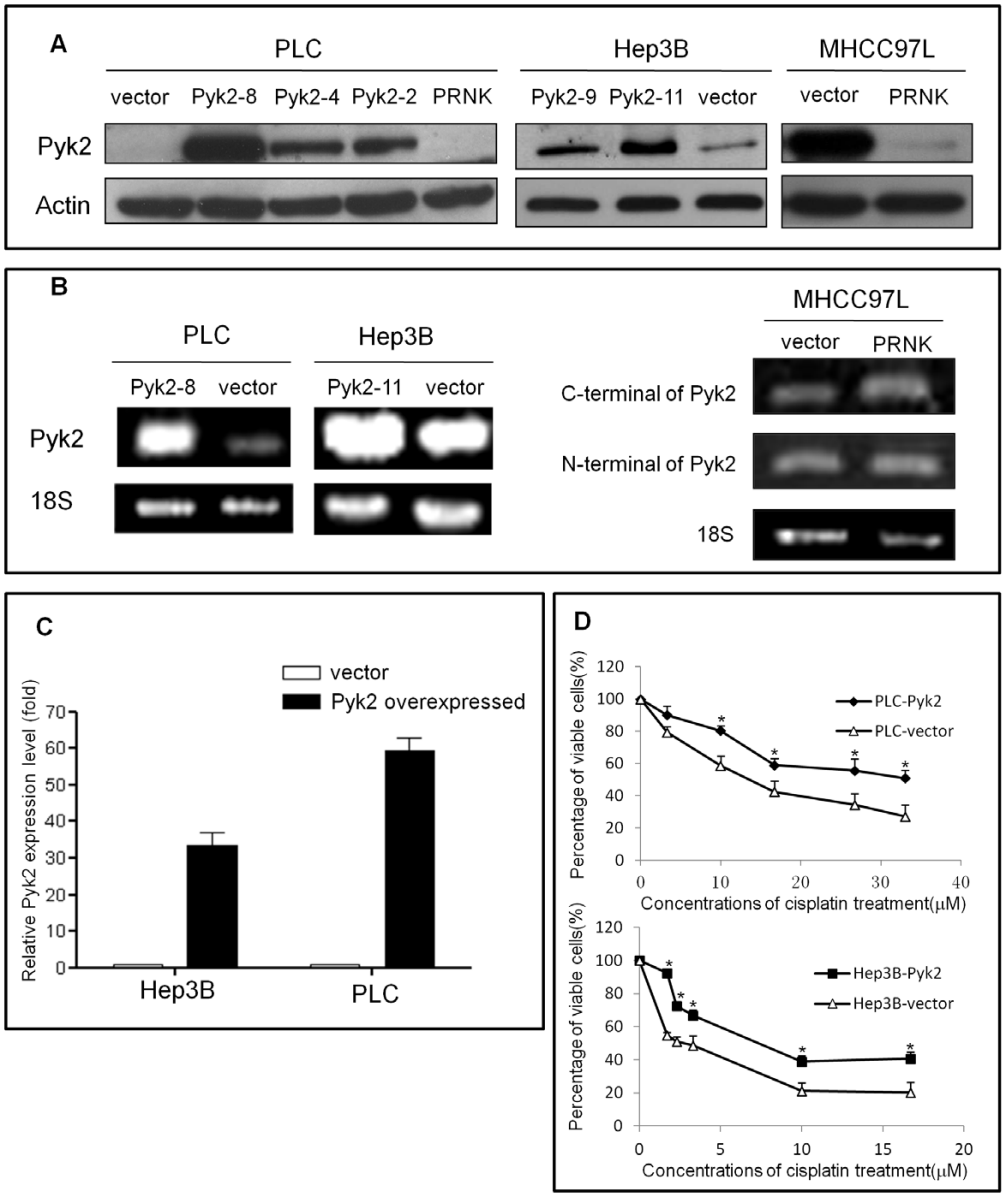
The establishment of Pyk2 overexpressed and suppressed stable transfectants. (A), Western blot analysis verified the protein expression level of Pyk2 in different stable transfectants (PLC, Hep3B and MHCC97L). Significantly higher Pyk2 expression was observed in PLC-Pyk2-8 and Hep3B-Pyk2-11. Pyk2 expression level was knocked down in MHCC97L by transfecting C-terminal of Pyk2. (B), RT-PCR demonstrated Pyk2 expression level in different stable transfectants at mRNA level. (C), Real time RT-PCR quantified Pyk2 expression level in these stable transfectants. (D), MTT assay showed that overexpression of Pyk2 promoted HCC cell proliferation rates upon increasing concentrations of cisplatin treatment.

We examined the expressions patterns of Pyk2 protein and mRNA in the stable transfectants (PLC, Hep3B and MHCC97L) by Western blot and RT-PCR. The expressions of Pyk2 mRNA and protein were significantly upregulated in Hep3B-Pyk2-11, PLC-Pyk2-8 and downregulated in MHCC97L-PRNK (Fig. 1A and 1B). Pyk2 mRNA were upregulated by 35-fold in Hep3B-Pyk2 and 60-fold in PLC-Pyk2 cells compared to Hep3B-vector and PLC-vector cells respectively (Fig. 1C).

### Overexpression of Pyk2 increased cisplatin-resistance of HCC cells

The result of MTT assay showed that the number of viable cells in Pyk2 overexpressing stable transfectants (Hep3B-Pyk2 and PLC-Pyk2) was significantly higher than the control groups (Hep3B-vector and PLC-vector) with administration of cisplatin (* *p*<0.05) (Fig. 1D).

**Figure 2:**
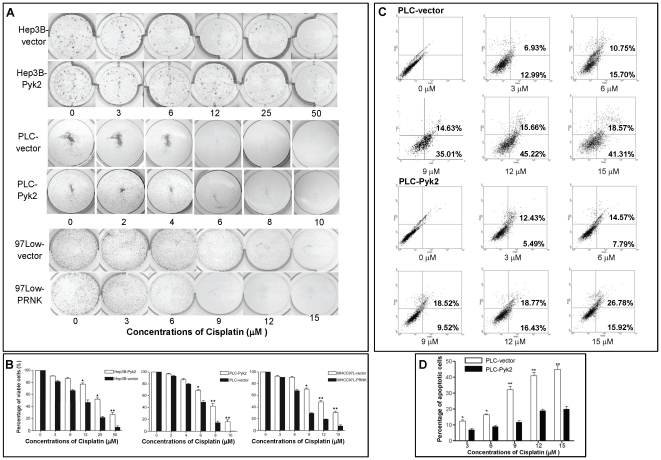
The effects of Pyk2 overexpression on HCC cell proliferation and apoptosis upon cisplatin treatment. (A), Colony Formation assay demonstrated that Pyk2 overexpressed stable transfectants had significantly higher cell proliferation rate upon increasing concentrations of cisplatin treatment. (B), Comparison of colony forming abilities among the HCC cell lines. * *p*<0.05; ** *p*<0.01. (C), Annexin-V-FLOUS assay showed that Pyk2 overexpression in PLC led to a reduction of apoptosis upon cisplatin treatment. (FL1 = Annexin-V-FLUOS, FL2 = prodium iodide; R1 = Living cells, R2 = apoptotic cells, R3 = necrotic cells) (D), Annexin-V-FLOUS assay showed that the percentage of apoptotic cells was significantly lower in PLC-Pyk2 by comparing with PLC-vector upon different concentrations of cisplatin treatment. * *p*<0.05; ** *p*<0.01.

In addition to the MTT assay, cell proliferation was evaluated by colony formation assay (Fig. 2A). The colony forming ability of Hep3B-Pyk2 cells was1.6-fold and 2.4-fold higher than Hep3B cells at 12 µM (79.6±2.5% *vs* 49.2±2.7%; *p* = 0.001) and 25 µM (52.9±4.0% *vs* 22.5±3.9%; *p* = 0.001) of Cisplatin (Fig. 2A). The difference of colony formatting ability reached to 6-fold at the 50 µM of cisplatin concentration (30.6±6.2% *vs* 5.2±2.1%; *p* = 0.001). The IC50 of cisplatin to Hep3B-Pyk2 transfectants was determined as 25 µM which was 2-fold higher than the Hep3B-vector transfectant (Fig. 2B).

In PLC cells, The colony forming ability of PLC-Pyk2 cells was1.4-fold and 1.6-fold higher than PLC cells at 6 µM (69.9±5.4% *vs* 50.8±5.5%; *p* = 0.013) and 8 µM (48.2±6.0% *vs* 17.8±3.7%; *p* = 0.002) of Cisplatin (Fig. 2A). The difference of colony formatting ability reached to 10-fold at the 10 µM of cisplatin concentration (18.6±3.5% *vs* 1.8±0.8%; *p* = 0.001). The IC50 of cisplatin to PLC-Pyk2 transfectants was determined as 6.3 µM which was 1.5-fold higher than the PLC-vector transfectant (Fig. 2B).

In MHCC97L cell lines with overexpressed Pyk2, transfection of PRNK significantly down-regulated its colony forming ability. There was a 2.7-fold, 2.5 fold and 4-fold decrease of colony forming ability as compared to the transfectants with empty vector at 9 µM (76.9±4.4 *vs* 28.8±2.2%; *p* = 0.001), 12 µM of cisplatin concentration (50.2±2.7 *vs* 19.8±2.1%; *p* = 0.001) and 25 µM of cisplatin concentration (36.9±4.4 *vs* 19.8±2.7%; *p* = 0.001). The IC50 of cisplatin to MHCC97L-PRNK transfectants was 5.5 µM which was 1.8-fold lower than the MHCC97L-vector transfectants (Fig. 2B). The cell viability (%) of each cell line under different concentrations of cisplatin was determined by colony formation assay and the results were presented in means±SD calculated from three independent experiments.

### Overexpression of Pyk2 reduced apoptosis of HCC cell lines upon cisplatin treatment

The effect of Pyk2 overexpression on cell apoptosis under the cisplatin environment was analyzed by propidium iodide and Annexin-V staining following by FACS analysis. The result demonstrated that PLC-Pyk2 exhibited a lower rate of apoptosis upon cisplatin environment than PLC-vector cells (Fig. 2C). When the concentration of cisplatin rose up to 9 µM, 12 µM and 15 µM, the percentage of apoptotic cell in PLC-vector was more than 2-fold of the PLC-Pyk2 cells (9 µM: 33.8±3.7% *vs* 11.0±1.5%, 12 µM: 42.3±4.8% *vs* 18.2±2.2%, *p* = 0.017; 15 µM: 45.9±5.1% *vs* 19.8±2.7%, *p*<0.01, Fig. 2D).

### Suppression of Pyk2 increased tumor necrosis/apoptosis upon cisplatin treatment

**Figure 3:**
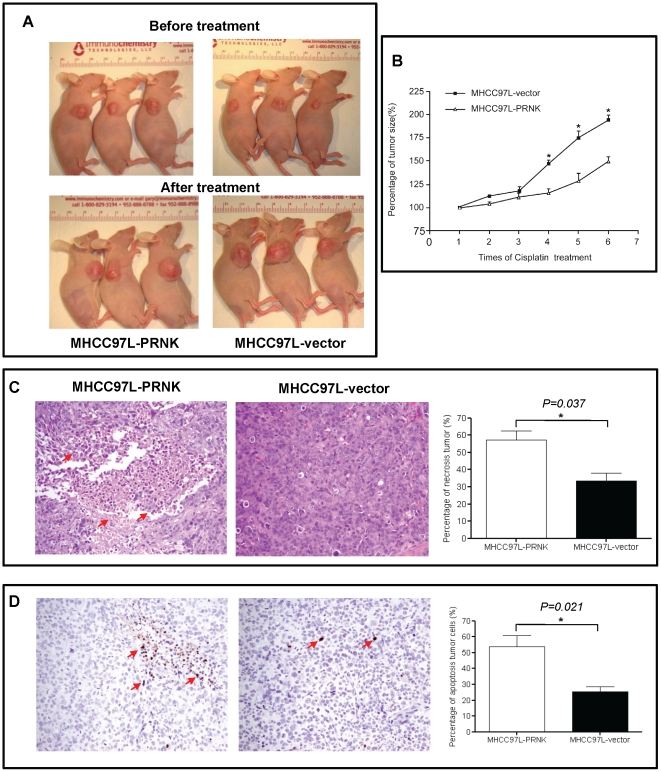
The establishment of subcutaneous xenograft nude mice model. (A), Liver tumor growth after cisplatin treatment. (B), Comparison of liver tumor growth * *p*<0.05 (C), H&E staining showed that tumor necrosis was more severe in MHCC97L-PRNK generated tumor (magnification ×40). By measuring the percentage of necrotic tumor at 5 different fields from 3 mice, more significant necrosis was observed in the MHCC97L-PRNK group. (D), TUNEL staining showed that tumor cell apoptosis was more severe in MHCC97L-PRNK generated tumor (magnification ×40). By counting the number of apoptotic particles at 5 different fields from 3 mice, more apoptotic cells were observed in MHCC97L-PRNK group.

We employed an ectopic xenograft model by subcutaneous injection of MHCC97L-vector and MHCC97L-PRNK cells into the nude mice. The result showed that the MHCC97L-PRNK group displayed a significantly reduced size of subcutaneous tumor compared to the MHCC97L-vector group after 6 times of cisplatin treatment (199.9±10.8 mm^3^ *vs* 138.9±14.6 mm^3^; *p* = 0.006, Fig. 3A). The growth rate of tumor in MHCC97L-PRNK was significantly slower than that in the MHCC97L-vector group (* *p*<0.05) (Fig. 3B). By measuring the percentage of necrosis at 5 different fields from 3 mice, higher percentage of necrotic cells was observed in the MHCC97L-PRNK group compared with MHCC97L-vector group (57.3±5.4% *vs* 32.8±4.9%, *p* = 0.037; Fig. 3C). By counting the number of apoptotic cells at 5 different fields from 3 mice, more apoptotic cells were observed in MHCC97L-PRNK group compared to the MHCC97L-vector group (53.7±6.9 *vs* 26.9±3.1, *p* = 0.021; Fig. 3D).

**Figure 4:**
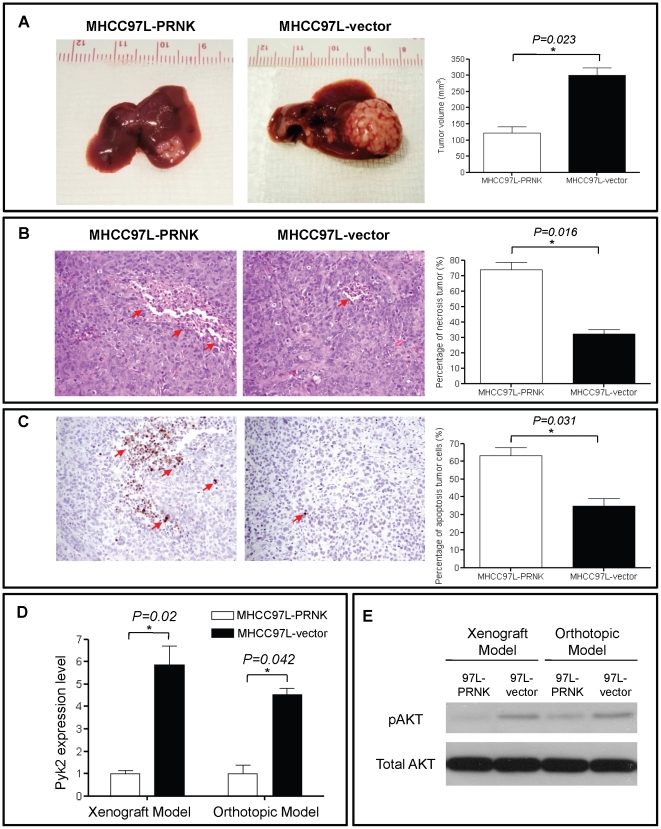
The establishment of orthotopic nude mice model. (A), After the cisplatin treatment, the tumor which generated by MHCC97L-vector is significantly larger than MHCC97L-PRNK group. (B), H&E staining showed that more significant necrosis was observed in the MHCC97L-PRNK group by measuring the percentage of necrosis at five different fields from 3 mice. * *p*<0.05. (C), TUNEL staining showed that tumor cell apoptosis was more severe in MHCC97L-PRNK generated tumor (magnification ×40) by measuring the percentage of apoptosis at five different fields from 3 mice. * *p*<0.05 (D), Real time RT-PCR illustrated that Pyk2 was overexpressed in MHCC97L-vector generated tumor in subcutaneous nude mice model and orthotopic model. * *p*<0.05. (E), phosphorylated Akt was also demonstrated to be overexpressed in MHCC97L-vector generated tumor in subcutaneous nude mice model and orthotopic model by western blot.

In the orthotopic xenograft model, tumor size in MHCC97L-PRNK was significantly reduced compared to the MHCC97L-vector group (MHCC97L-vector: 301.7±16.7 mm^3^; MHCC97L-PRNK: 120.6±10.3 mm^3^, *p* = 0.023, Fig. 4A). By measuring the percentage of necrotic tumor cells at 5 different fields from 3 mice, higher percentage of necrotic cells was observed in the MHCC97L-PRNK group compared to the MHCC97L-vector group (73.1±4.4% *vs* 31.3±4.3%, *p* = 0.016; Fig. 4B). Moreover, more apoptotic cells were observed in MHCC97L-PRNK group than the MHCC97L-vector group (62.3±5.1 *vs* 34.9±6.0%, *p* = 0.031; Fig. 4C). The expression level of Pyk2 mRNA in tumor tissues was further confirmed by RT-PCR to be downregulated in MHCC97L-PRNK group in both ectopic and orthotopic xenograft models (Fig. 4D). Moreover, phosphorylated Akt was also demonstrated to be overexpressed in MHCC97L-vector generated tumor in subcutaneous nude mice model and orthotopic model by western blot (Fig. 4E).These results indicated that Pyk2 overexpression had a significant effect on tumor growth by activation of pAkt, as well as inhibition of tumor necrosis and apoptosis upon cisplatin treatment.

### Overexpression of Pyk2 differentially regulated multidrug resistant genes in HCC cells

**Figure 5:**
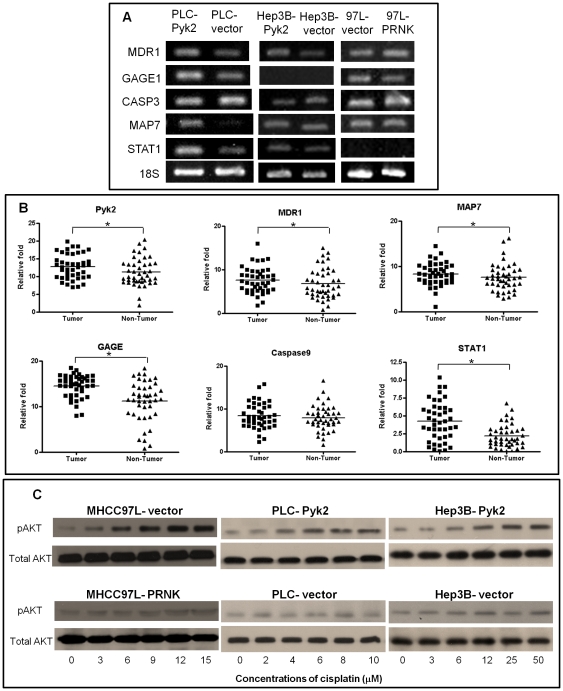
Exploration of down-regulate signaling pathway and the underlying mechanism. (A), RT-PCR verified that MDR1, GAGE1, MAP7, and STAT1 have positive correlations the expression level of Pyk2 in PLC, Hep3B and MHCC97L cell lines. (B), Expression level of Pyk2, MDR1, GAGE1, MAP7, and STAT1 was higher in HCC samples. (C), Dose-dependent western bolt assay demonstrated that Phosph-AKT expression increasing by following the increasing concentrations of cisplatin treatment in Pyk2 overexpressed stable transfectants (MHCC97L-vector, PLC-Pyk2, Hep3B-Pyk2).

The mechanisms of Pyk2 overexpression related cisplatin resistance in HCC were investigated by cDNA microarray analysis. After comparing the gene profiles of PLC-Pyk2-8 and PLC-vector cells, 4,322 upregulated and 1,891 downregulated genes were found in PLC-Pyk2-8 cells. The functions of most upregulated genes were identified to be involved in signal transduction (16%), transcription (14%), cell defense response (8%), immune response (8%), cell cycle (8%), cell proliferation (8%), cell adhesion (6%), apoptosis (6%), and cell growth (4%). Among those downregulated genes, most functions were related to nucleic acid binding (29%), transcription factor activity (17%), protein binding (15%), apoptosis (10%), signal transducer activity (5%), negative regulation of cell proliferation (5%), and cell cycle (5%) (Fig. S1). Fifty-five upregulated genes and 50 downregulated genes with more than 2-fold changes were identified (Table S1). These target genes were narrowed down by their correlation with cisplatin resistance and HCC invasiveness subsequently. G antigen 1 (GAGE1), multidrug resistant gene-1 (MDR1), signal transducer and activator of transcription (STAT1), Caspase9 and microtubule-associated protein 7 (MAP7) were chosen for further validation by RT-PCR. Most of them could also be verified to be differentially expressed in Hep3B and MHCC97L cell lines (Fig. 5A).

To examine the expression correlation between Pyk2 and these genes in HCC patients, the expression profiles of MDR1, GAGE1, Caspase9, MAP7 and STAT1 mRNA in 43 pairs of tumor and adjacent non-tumor liver tissues of HCC patients underwent hepatectomy were analyzed. Pyk2, MDR1, GAGE1, MAP7 and STAT1 were found to be overexpressed in more than 60% of the tumor liver tissues compared to the adjacent non-tumor liver tissues (*p<0.05) (Table 2 and Fig. 5B). The expressions patterns of MDR1, GAGE1, MAP7 and STAT1 were significantly correlated with Pyk2 expression in HCC patients (*p*<0.01).

**Table 2:** Expression level of Pyk2, MDR1, GAGE, MAP7 and STAT1 in 43 pairs of clinical samples.

Gene name	Suppression	Over-expression	Highly over-expression*	No difference
Pyk2	11.6%(5/43)	65.1%(28/43)	46.5%(20/43)	23.2%(10/43)
MDR1	9.3%(4/43)	60.5%(26/43)	41.8%(18/43)	30.2%(13/43)
Casp9	30.2%(13/43)	46.6%(20/43)	44.2%(19/43)	23.2%(10/43)
GAGE	13.9%(6/43)	79.1% (34/43)	65.1%(28/43)	7.0%(3/43)
MAP7	18.6%(8/43)	62.8% (27/43)	44.2%(19/43)	18.6%(8/43)
STAT1	27.9%(12/43)	65.1%(28/43)	46.5%(20/43)	7.0%(3/43)

*More than 2 fold difference in the tumor tissue.

### Overexpression of Pyk2 activated Akt phosphorylation upon cisplatin treatment

Western blot analysis was employed to investigate the role of Pyk2 on AKT signaling pathway of HCC cells in responding to cisplatin treatment. Akt phosphorylation was activated by cisplatin in a dose-dependent manner in the HCC cell lines with Pyk2 over-expression (MHCC97L-vector, PLC-Pyk2 and Hep3B-Pyk2). While in control groups (MHCC97L-PRNK, PLC-vector and Hep3B-vector), Phospho-AKT was slightly increased in different concentrations of cisplatin (Fig. 5C).

## Discussion

Although cisplatin is a common therapeutic agent used for chemotherapy in HCC patients, its curative effect is significantly limited due to chemoresistance of HCC [31]. Acquisition of cisplatin resistance during chemotherapy becomes a vital factor to cancer mortality and remains a major clinical obstacle. Cisplatin is a cytotoxic compound that causes apoptosis via DNA damage by formation of interstrand or intrastrand adducts[32]. The cytotoxic effect of cisplatin is a complex process that leads to activation of several pathways organized in a large network. Blockade of any of the signaling involved may result in reduction of chemosensitivity. In general, mechanisms inhibiting propagation of the DNA damage signal to the apoptosis include loss of damage recognition, overexpression of HER-2/neu, activation of the PI3-K/AKT pathway, loss of p53 function, overexpression of anti-apoptotic bcl-2, and interference in caspase activation [33]. Upon disregulations of these genes, cisplatin resistance could be induced by several aspects including reduction of intracellular drug accumulation, increased inactivation by the thiol-containing molecules, increasing of DNA damage repair and inhibition of apoptosis. However, the mechanisms of cisplatin resistance in HCC and the mechanism underlying clinical cisplatin resistance remain poorly understood. By fully understanding these mechanisms, we may offer a promising approach to mediate cisplatin sensitivity and the possibility of developing a personalized therapeutic strategy.

In most studies of chemoresistance, tumor cells with chemoresistance were selected by stepwise exposure to increasing concentrations of chemotherapeutic drugs[34]. After comparing the difference of gene expression profiles between resistant and normal cells, several potential target genes were selected for further study. Therefore the gene overexpressed in a well-established resistant cell line might be a consequence of the acquired resistance rather than the direct cause leading to chemoresistance. In this study, Pyk2-overexpressed stable transfectants were established instead. Afterwards, Pyk2 overexpression-induced cisplatin resistance was observed in both the *in vitro* and *in vivo* studies. cDNA microarray was performed to explore the mechanism of Pyk2 overexpression-induced cisplatin resistance subsequently. The novelty of this approach may allow it to have the advantage in identifying the direct role of Pyk2 in cisplatin resistance in HCC.

Pyk2 inherits 60% from the central kinase domain of FAK and has a similar domain structure with 40% identities in the FAT domain regions[14]. FAK was also confirmed to be a key factor involved in liver tumor progression and have prognostic significance for HCC[35]. We have demonstrated that overexpression of Pyk2 can induce the upregulation of c-Src and ERK[23]. Interestingly, the FAT domain also contains Tyr881, which serves as a binding site for the adaptor Grb2 and linkage to the MAP kinase signaling pathway when phosphorylated by Src [36]. It happens to be a key event to activate PI3-K/AKT signaling pathway and induce cisplatin resistance subsequently.[37], [38] Based on such information, we hypothesized that overexpression of Pyk2 may play a crucial part in cisplatin resistance in HCC.

Our results demonstrated that overexpression of Pyk2 could contribute cisplatin resistance to HCC cells by promoting proliferation and reducing apoptosis. Moreover, suppression of Pyk2 by PRNK in MHCC97L cells could reduce cisplatin resistance of the cells by significantly suppressing its proliferation. Furthermore, suppression of Pyk2 by PRNK significantly inhibited the orthotopic tumor growth after cisplatin treatment compared to the control group by induction of tumor cell necrosis and apoptosis. The above data suggested that overexpression of Pyk2 may be one of the mechanisms contributing to drug resistance in HCC.

There were more than 6,000 differential genes detected in Pyk2 overexpressing HCC cells and those differential genes were found to be involved in different biological functions, indicating that common upregulation of Pyk2 in HCC may contribute to deregulation of a large number of genes.

The expressions of several drug-resistant genes including MDR1, GAGE, STAT1, and MAP7 were found to be overexpressed in three Pyk2 overexpressing HCC cell lines and in tumor liver tissues of HCC patients. Moreover, their expression levels also showed significant positive correlations with Pyk2 expression in HCC patients, indicating that upregulations of drug resistant genes may be a possible mechanism of Pyk2-induced drug resistance in HCC.

The GAGE1 cDNA contains a 143-bp insertion located in the coding sequence near the termination codon, which is absent from the other cDNAs. Interestingly, its expression could be detected in many types of tumor tissues. However, GAGE expression could be observed in none of the non-tumor tissues except for testis[39]. A recent study pointed out that GAGE family members are expressed in medulloblastoma cells and specimens, and the inhibition of GAGE genes in MB cells can sensitize them to certain chemotherapeutic agents such as cisplatin[40]. The MDR1 encodes P-glycoprotein (P-gp) and plays an important role in mediating multidrug resistance to chemotherapeutic agents. In cancer, P-gp overexpression is associated with a poor response to chemotherapy[41]. Overexpression of MDR1 was observed in many types of cancer such as HCC[42] and non-small-cell lung cancer[43]. In HCC, it was proven that amplification of MDR1 mRNA is probably the main mechanism underlying acquired doxorubicin resistance[44]. The protein encoded by STAT1 is a member of the STAT protein family. In HCC cell lines, knockdown of STAT1 or Janus kinase1 suppressed the dephosphorylation of both ERK and MEK and diminished the cell growth[45]. Therefore, upregulation of STAT1 may promote cell proliferation even under the cisplatin environment. Additionally, STAT1 belongs to the IFN-related DNA damage resistance signature which is proven to be associated with resistance to chemotherapy across different cancer cell lines[46]. The product of MAP7 is a microtubule-associated protein predominantly expressed in cells of an epithelial origin. Microtubule-associated proteins are thought to be involved in microtubule dynamics, which is essential for cell polarization and differentiation. Some studies indicated that determination of mRNA levels of MAP7 may be useful for prediction of the success of neoadjuvant chemotherapy in Barrett's carcinoma[47]. Since these drug-resistance related genes were up-regulated by Pyk2, it may suggested that Pyk2 overexpression could be a crucial factor in cisplatin resistance in HCC. To further study the underlying mechanism of Pyk2 induced cisplatin resistance, we need to find out which pathways were activated by overexpression of Pyk2 in HCC.

Many studies suggested PI3K/AKT/mTOR survival pathway plays an important role in cisplatin resistance in ovarian cancer[48], lung cancer[49] and breast cancer[50]. Moreover, the inhibitor of AKT was proven to have significant effect on increasing cisplatin sensitivity in these cancers. Therefore, AKT was considered to be a potential target to overcome cisplatin resistance. In HCC, PI3K/AKT pathway was also demonstrated to have significant correlations with cisplatin resistance[37]. The blockade of Akt/HIF-1alpha/PDGF-BB autocrine signaling can enhance the chemosensitivity of liver cancer cells and tumorigenic hepatic progenitor cells under hypoxic conditions[38]. Based on these information, we hypothesized Pyk2 may induce cisplatin resistance in HCC by activation of PI3K/AKT pathway. To confirm our hypothesis, a dosage-dependent assay was preformed to demonstrate the correlation between Pyk2 and AKT activation. The result showed that phosphorylated AKT expression level was gradually increased by increased concentrations of cisplatin in Pyk2 overexpressed HCC cell lines (MHCC97L-vector, PLC-Pyk2 and Hep3B-Pyk2). However, this dose-dependent response could not be observed in HCC cells with lower Pyk2 expression (MHCC97L-PRNK, PLC-vector and Hep3B-vector) (Fig. 5C). This result indicated that when HCC cell line was treated with cisplatin, overexpression of Pyk2 could induce the upregulation of AKT in a cisplatin dose-dependent manner. Moreover, the activation of pAkt was also well correlated with higher expression of Pyk2 and less tumor apoptosis in our *in vivo* models underwent cisplatin treatment.

In summary, our results showed that overexpression of Pyk2 is associated with acquired cisplatin resistance in HCC through promoting cell proliferation, reducing apoptosis, activation of AKT pathways and upregulation of drug-resistant genes. Pyk2 may not only be applied as a potential predictive marker of chemoresistance for platinum-based chemotherapy in HCC patients, but also be a novel therapeutic target in HCC.

## Supporting Information

Figure S1 **Gene profiles of two stable transfectants were compared between PLC-Pyk2–8 and PLC-vector.**
(TIFF)

Table S1 **Gene profiles regulated by Pyk2.**
(DOC)
