# Supplementary figures and images for: The Role of Proline Rich Tyrosine Kinase 2 (Pyk2) on Cisplatin Resistance in Hepatocellular Carcinoma
Source: PLoS One. 2011 Nov 9;6(11):e27362. doi: 10.1371/journal.pone.0027362 (PMC3212555; doi:10.1371/journal.pone.0027362)

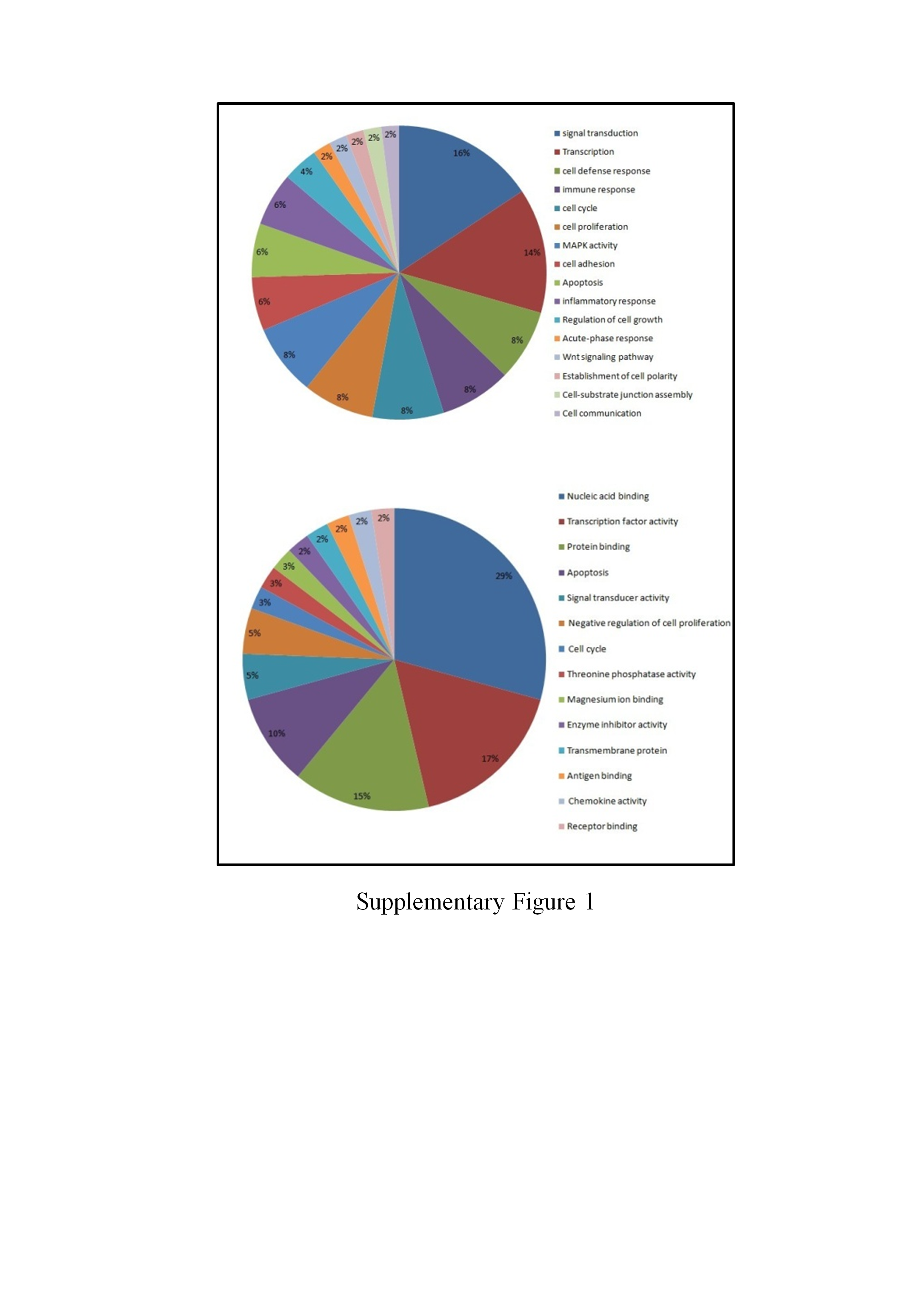

Supplement: Figure S1 — Gene profiles of two stable transfectants were compared between PLC-Pyk2–8 and PLC-vector. (TIFF) [file pone.0027362.s001.tiff]
