# Supplementary material for: The Role of Proline Rich Tyrosine Kinase 2 (Pyk2) on Cisplatin Resistance in Hepatocellular Carcinoma
Source: PLoS One. 2011 Nov 9;6(11):e27362. doi: 10.1371/journal.pone.0027362 (PMC3212555; doi:10.1371/journal.pone.0027362)
Supplement: Table S1 — Gene profiles regulated by Pyk2. (DOC) [file pone.0027362.s002.doc]

Table S1 Gene profiles regulated by Pyk2

| **Regulation** | **Fold Change** | **Gene Symbol** | **Gene Ontology Biological Process** |
| --- | --- | --- | --- |
| UP | 1465.16 | GAGE3 | cellular defense response |
| UP | 305.5 | GAGE12B | cellular defense response |
| UP | 298.12 | GAGE12F | cellular defense response |
| UP | 282.26 | GAGE1 | cellular defense response |
| UP | 121.02 | VCAN | cell adhesion |
| UP | 35.48 | CD55 | immune response |
| UP | 24 | CD163 | immune response |
| UP | 3.12 | MDR1 | response to drug |
| UP | 17.29 | ABCB1 | response to drug |
| UP | 12.44 | FOXO3 | regulation of transcription |
| UP | 2.3 | FOXR2 | regulation of transcription |
| UP | 8.26 | IL28RA | regulation of cell proliferation |
| UP | 3.1 | IL2RB | signal transduction |
| UP | 4.34 | IL11 | regulation of cell proliferation |
| UP | 2.1 | IL18 | regulation of cell adhesion |
| UP | 2.2 | IL1R2 | immune response |
| UP | 2.6 | IL17D | inflammatory response |
| UP | 13.8 | TIMP2 | regulation of cell proliferation |
| UP | 8.99 | TIMP4 | regulation of cell proliferation |
| UP | 5.7 | FGFR13 | MAPKKK cascade |
| UP | 22.89 | FGF13 | activation of MAPKK activity |
| UP | 9.4 | FGF14 | signal transduction |
| UP | 4.6 | FGF2 | activation of MAPKK activity |
| UP | 5.13 | S100P | endothelial cell migration |
| UP | 4.3 | S100A8 | inflammatory response |
| UP | 9.4 | S100A9 | inflammatory response |
| UP | 2.1 | S100A10 | signal transduction |
| UP | 6.99 | CAV1 | inactivation of MAPK activity |
| UP | 5.66 | CCNA1 | Regulation of cell cycle |
| UP | 3.1 | CCNG2 | Cell cycle check point |
| UP | 2.9 | CXCL1 | Chemotaxis |
| UP | 2.5 | CXCL3 | Chemotaxis |
| UP | 5.56 | CXCL11 | Chemotaxis |
| UP | 2.04 | SMAD4 | Transcription |
| UP | 2.76 | SMAD6 | Transcription |
| UP | 2.06 | SMAD9 | Transcription |
| UP | 5.17 | IGFBP3 | Regulation of cell growth |
| UP | 2.1 | IGFBP1 | Regulation of cell growth |
| UP | 3.93 | IGF1R | Signal transduction |

|  |  |  |  |
| --- | --- | --- | --- |
| **Regulation** | **Fold Change** | **Gene Symbol** | **Gene Ontology Biological Process** |
| UP | 4.64 | ITGB4 | Cell communication |
| UP | 2.1 | ITGB3 | Cell-substrate junction assembly |
| UP | 3.03 | LAMB1 | Cell adhesion |
| UP | 2.57 | STAT1 | Regulation of transduction |
| UP | 3.27 | STAT4 | Regulation of transduction |
| UP | 4.35 | LTBR | Apoptosis |
| UP | 3.01 | IFI16 | Regulation of transduction |
| UP | 2.51 | NOTCH1 | Transcription |
| UP | 2.49 | PECAM1 | Cell motility |
| UP | 2.44 | ARHGAP9 | Signal transduction |
| UP | 2.43 | IER3 | Apoptosis |
| UP | 2.3 | CDK2 | G2/M transition of mitotic cell cycle |
| UP | 2 | CDC25B | M phase of mitotic cell cycle |
| UP | 10.34 | WNT10A | Wnt signaling pathway |
| UP | 32.34 | MAP7 | Establishment of cell polarity |
| UP | 4.87 | FN1 | Acute-phase response |
| Down | 2.13 | CASP9 | Apoptosis |
| Down | 2.12 | CAMTA1 | Transcription |
| Down | 34.6 | TCEAL3 | Transcription |
| Down | 16.5 | EGR4 | Nucleic acid binding |
| Down | 14.9 | RASGRP3 | Signal transducer activity |
| Down | 9.4 | CYCS | Threonine phosphatase activity |
| Down | 6.16 | ANGPTL4 | Enzyme inhibitor activity |
| Down | 4.54 | FZD6 | Signal transducer activity |
| Down | 4.4 | FGF18 | Growth factor activity |
| Down | 4.39 | CXCL5 | Chemokine activity |
| Down | 3.85 | BAX | Apoptosis |
| Down | 3.83 | EGR1 | Transcription factor activity |
| Down | 3.65 | CLDN2 | Structural molecule activity |
| Down | 3.47 | IGHG1 | Antigen binding |
| Down | 3.36 | JAK1 | Nucleotide binding |
| Down | 3.18 | LRRCC1 | Protein binding |
| Down | 3.18 | RPS6KA5 | Nucleotide binding |
| Down | 3.17 | ZCCHC10 | Nucleotide binding |
| Down | 3.09 | CMBL | Protein binding |
| Down | 3.05 | PSMG4 | Transcription activator activity |
| Down | 3.01 | MGC16385 | DNA binding |
| Down | 3 | ZNF621 | Nucleic acid binding |
| Down | 3 | GPNMB | Negative regulation of cell proliferation |
| Down | 2.97 | FGF18 | Growth factor activity |
| Down | 2.96 | ARHGEF7 | Nucleotide activity |
| Down | 2.91 | FERMT2 | Protein binding |
|  |  |  |  |
| **Regulation** | **Fold Change** | **Gene Symbol** | **Gene Ontology Biological Process** |
| Down | 2.89 | OSTF1 | Protein binding |
| Down | 2.89 | TMEM144 | Transmembrane protein |
| Down | 2.88 | OSGIN1 | Growth factor activity |
| Down | 2.88 | RNF12 | Transcription corepressor activity |
| Down | 2.85 | SNCA | Nucleic acid binding |
| Down | 2.81 | SFRP4 | Protein binding |
| Down | 2.8 | CYCS | Threonine phosphatase activity |
| Down | 2.72 | TCEAL1 | Transcription factor activity |
| Down | 2.7 | TNFSF15 | Receptor binding |
| Down | 2.65 | MCTS1 | RNA binding |
| Down | 2.67 | DBF4B | Nucleic acid binding |
| Down | 2.67 | PARD6A | Apoptosis |
| Down | 2.67 | EGR1 | Transcription factor activity |
| Down | 2.63 | NUDT4 | Magnesium ion binding |
| Down | 2.52 | DAB2 | protein binding |
| Down | 2.52 | OR2C1 | Signal transducer activity |
| Down | 2.46 | MAP9 | Cell cycle |
| Down | 2.38 | TM2D1 | Apoptosis |
| Down | 2.32 | TSC22D3 | Transcription factor activity |
| Down | 2.19 | RARRES3 | Negative regulation of cell proliferation |
| Down | 2.07 | MGC16385 | Transcription activator activity |
| Down | 2.03 | ZNF621 | DNA binding |
| Down | 2.02 | GPNMB | Nucleic acid binding |
| Down | 2 | RARRES3 | Negative regulation of cell proliferation |
